# Terminal nucleotidyl transferases (TENTs) in mammalian RNA metabolism

**DOI:** 10.1098/rstb.2018.0162

**Published:** 2018-11-05

**Authors:** Zbigniew Warkocki, Vladyslava Liudkovska, Olga Gewartowska, Seweryn Mroczek, Andrzej Dziembowski

**Affiliations:** 1 Department of RNA Metabolism, Institute of Bioorganic Chemistry, Polish Academy of Sciences, Noskowskiego 12/14, Poznan, Poland; 2 Laboratory of RNA Biology and Functional Genomics, Institute of Biochemistry and Biophysics, Polish Academy of Sciences, Pawinskiego 5a, 02-106 Warsaw, Poland; 3 Institute of Genetics and Biotechnology, Faculty of Biology, University of Warsaw, Pawinskiego 5a, 02-106 Warsaw, Poland

**Keywords:** TENT, non-canonical polyadenylation, RNA uridylation, TUTase, RNA stability, RNA metabolism

## Abstract

In eukaryotes, almost all RNA species are processed at their 3′ ends and most mRNAs are polyadenylated in the nucleus by canonical poly(A) polymerases. In recent years, several terminal nucleotidyl transferases (TENTs) including non-canonical poly(A) polymerases (ncPAPs) and terminal uridyl transferases (TUTases) have been discovered. In contrast to canonical polymerases, TENTs' functions are more diverse; some, especially TUTases, induce RNA decay while others, such as cytoplasmic ncPAPs, activate translationally dormant deadenylated mRNAs. The mammalian genome encodes 11 different TENTs. This review summarizes the current knowledge about the functions and mechanisms of action of these enzymes.

This article is part of the theme issue ‘5′ and 3′ modifications controlling RNA degradation’.

## 1. Introduction

**Figure 1.**
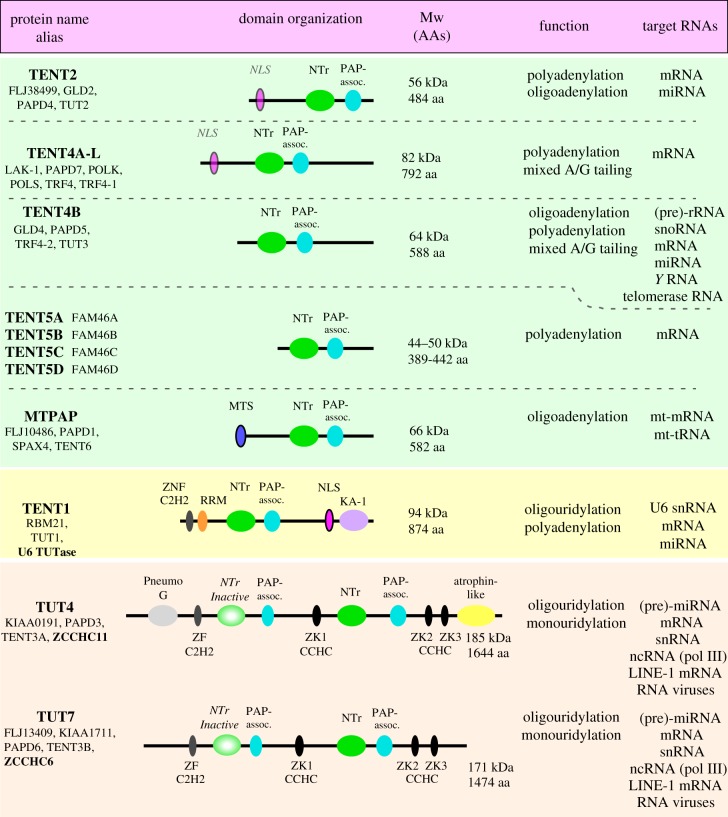
A display of mammalian TENTs. The display summarizes major facts about 11 mammalian TENTs. The enzymes fall within two major classes: poly(A) polymerases (highlighted in green) and poly(U) polymerases (highlighted in yellow and pink). Their homologues can be further grouped into seven families based on their phylogenetic conservation (separated by dashed lines and/or coloured background). The display states protein names according to currently recommended terminology, the multiple aliases, protein molecular weights (in kDa) and number of amino acids within the canonical isoform (after uniprot.org), activities and targeted RNA types. Additionally, a cartoon representation of each protein (or a consensus representation for the TENT5 proteins) is provided with indicated domains that are colour-coded and labelled as follows (in alphabetical order): KA-1—kinase associated domain (in TENT1), MTS—mitochondria targeting signal/peptide, NLS—nuclear localization signal, NTr—catalytic nucleotidyltransferase domain (or an inactive domain), PAP-associated domain, Pneumo G and atrophin-like domains in TUT4, RRM—RNA recognition motif, ZNF—zinc finger domain of either C2H2 or CCHC types. snRNA, small nuclear RNA.

Eukaryotic gene expression pathways are very complex and regulated at multiple levels. Essentially all RNAs are processed at their 3′ ends and most coding mRNAs, as well as some non-coding RNAs (ncRNAs), are polyadenylated in the nucleus by canonical poly(A) polymerases at the end of transcription. In recent years, 11 TErminal NucleotidylTransferases (TENTs) have been discovered (figure 1) [1–3]. TENTs contain a particular catalytic fold that is defined by InterPro as a nucleotidyl transferase domain (IPR005835 or PF00483) belonging to the DNA polymerase β (Pol β) superfamily that could catalyse non-templated nucleotide additions to RNA 3′ ends [1,4,5]. Based on their substrate preference towards adenosine monophosphate (AMP) or uridine monophosphate (UMP) incorporation, human TENTs are divided into non-canonical poly(A) polymerases (ncPAPs) and terminal uridyl transferases (TUTases), while phylogenetic analysis groups them into seven families [4,5]. TENTs share a common two-metal ion catalytic mechanism involving a highly conserved triad of aspartate residues in their catalytic helix-turn motif [6–10]. TENTs are not restricted to the nucleus and have specific regulatory roles also in the cytoplasm and mitochondria. Indeed, their functions are quite diverse and range from RNA maturation and decay to activation of translationally dormant deadenylated mRNAs. The exploration of TENTs' impact on the regulation of gene expression has become a rapidly growing field of research. Recently, the nomenclature of human TENTs and their orthologues across vertebrates has been updated and is presented in figure 1 and used throughout the paper. In this review, we discuss the current state of knowledge regarding mammalian TENTs.

## 2. ncPAPs and adenylation

The best-known example of non-templated adenylation is at 3′ ends of the vast majority of protein-coding mRNAs. This phenomenon, first realized in the early 1970s, is represented by the activity of two nuclear ‘canonical’ poly(A) polymerases—PAPα and PAPγ, and a great number of other auxiliary, structural and enzymatic factors as reviewed in detail elsewhere [11–13]. The primary role of the poly(A) tail is to protect an mRNA's 3′ end from degradation, thus contributing to its stability and increased translation rate [14]. The existence of ncPAPs came with the discovery of cytoplasmic polyadenylation regulating the timing of mRNA translation and stability in developing *Caenorhabditis elegans* and *Xenopus laevis* embryos by TENT2 protein (GLD2) [15,16]. In this part of the review, we describe the eight mammalian ncPAPs, including some crucial findings about their homologues in other organisms.

### (a) TENT2, also known as FLJ38499, GLD2, PAPD4, TUT2

The bulk of the data on TENT2 came from studies in non-mammalian species, including *C. elegans*, *X. laevis* and *Drosophila melanogaster*. In these organisms, TENT2 was first described as a ncPAP with a key role in translational activation of a subset of cytoplasmic mRNAs through elongation of their poly(A) tails [15–17]. The functional regulation of mRNA translation in gametes and early embryos in these organisms is accomplished by highly regulated polyadenylation–deadenylation cycles that, besides TENT2, involve several other factors [16–27]. There are comprehensive reviews on the role of TENT2 in gametogenesis and early development in non-mammalian species [28–30].

On the basis of the discoveries in *C. elegans* and *X. laevis* it seemed likely that TENT2 is involved in gametogenesis and early embryo development in mammals. This was further supported by the heterologous translation activator activity of human TENT2 tethered to a reporter mRNA and injected into *X. laevis* oocytes [31]. In line with this hypothesis, knock-down or overexpression of TENT2 in mice oocytes results in a delay of their maturation and frequent arrest in metaphase I [32]. Surprisingly, TENT2-deficient mice of both sexes are fertile and do not demonstrate any gross phenotype. The maturation of oocytes is normal and the length of poly(A) tails of the reporter mRNA is altered neither in germline nor in somatic cells [33]. This raises a possibility that in mammalian early embryos other yet unidentified TENT protein(s) might be involved in poly(A) length regulation [34] or that other processes like regulation of mRNA decay by uridylation-mediated mechanisms (see §3b on TUTases) play decisive roles [35].

On an organismal level, besides a possible auxiliary role in early embryo development, TENT2 may also be necessary for long-term memory formation in mice as it is expressed in the hippocampus and co-localizes with proteins involved in synaptic plasticity, such as Pumilio and CPEB1 [17]. TENT2 was shown to polyadenylate GluN2A mRNA encoding a subunit of the postsynaptic N-methyl-d-aspartate receptor, crucial for synaptic plasticity in rat hippocampal neurons [36]. Furthermore, TENT2 polyadenylates hnRNPA1, p27kip1 and β-catenin mRNAs in human 293T cells [37], which might play some role in cell cycle regulation in agreement with some earlier findings in *X. laevis* [38]. The latter mRNAs are specifically targeted for polyadenylation by QKI-7 protein, which first binds the mRNAs and then recruits TENT2 to execute polyadenylation. Polyadenylation by TENT2 stabilizes mRNA and augments their translation.

Some further confirmed roles of TENT2 in mammals are in miRNA regulation. TENT2 is responsible for monoadenylation of certain mature miRNAs like a liver-specific *miRNA-122*, involved in the regulation of fatty acid homeostasis. The miRNA was found to be 3′ monoadenylated both in human hepatocytes and in mice livers [39]. Since in *TENT2* knock-out mice the *miRNA-122* level is significantly lower than in wild-type mice, it has been suggested that monoadenylation of miRNA by TENT2 enhances its stability [39]. In line with these findings is the observation that TENT2 depletion in human fibroblast cell line causes a significant reduction of a fraction of monoadenylated miRNAs [40]. Furthermore, the stabilizing effect of monoadenylation on miRNA depends on the nucleotide composition within the miRNA 3′ region [40]. TENT2 also acts as a poly(A) polymerase on miRNAs in mouse hippocampal neurons, but its deletion has no detectable effect on mice behaviour [41].

There is a certain controversy regarding the involvement of TENT2 in the monouridylation and oligouridylation of pre-miRNA, especially of the so-called group II miRNA family including most of the *let-7* miRNAs. Essentially, TENT2 was suggested to participate in this process redundantly with two other confirmed human terminal uridyltransferases: TUT4 and TUT7 [42,43]. While TENT2 purified from human cells uridylated *pre-let-7* pre-miRNA [42,43], a recombinant protein purified from *Escherichia coli* showed superior specificity towards ATP in comparison to UTP with an enzymatic efficiency (*k*_cat_/*K*_m_) roughly two orders of magnitude higher for ATP than for UTP [44]. Interestingly, a single histidine insertion at position 440 of human TENT2 results in a switch from an ATP to a UTP preference of the protein [44]. These data, together with a lack of solid evidence for an *in vivo* uridylating activity of TENT2, suggest that TENT2 is a *bona fide* ncPAP and not a TUTase.

### (b) TENT4A, also known as LAK-1, PAPD7, POLK, POLS, TRF4 and TRF4-1

TENT4A is a human orthologue of the yeast Trf4p protein. Trf4p is a key subunit of the so-called TRAMP complex, within which it specifies mRNAs for surveillance and turnover by the nuclear exosome 3′–5′ ribonuclease complex [45,46], reviewed in [47,48]. However, TENT4A has not been identified as a component of the human TRAMP complex [49].

TENT4A was shown to exist in two isoforms: TENT4A short (S) and TENT4A long (L). The latter possesses a longer N-terminal region and seems to be the predominant isoform in the cell [50]. Although both isoforms contain a nucleotidyltransferase domain, only TENT4A L is able to add poly(A) tails when tethered to an RNA. TENT4A L is mainly localized in the nucleus but is excluded from the nucleolus. Interestingly, TENT4A S is evenly distributed throughout the cell, whereas only a small fraction of TENT4A L could be found in the cytoplasm. Further analysis revealed that the N-terminal region is crucial not only for nucleotidyltransferase activity but also for the nuclear localization of TENT4A L [50]. In fact, on the basis of ribosome profiling [51] and recent experimental work by Lim *et al*. [52] it has been ascertained that TENT4A L possesses 20 amino acids more on its N-terminus than previously annotated [50]. These, and an additional 10 N-terminal AAs show strong conservation with the N-terminus of another human Trf4p homologue—TENT4B [52]. There are some suggestions that TENT4A may be involved in pre-mRNA maturation in the nucleoplasm, as TENT4A S was shown to interact with a non-nucleolar protein PRPF31, which is necessary for U4/U6-U5 tri-snRNP formation [53]. Interestingly, TENT4B has also been reported to interact with a subset of splicing factors, among others with PRPF31 [49]. Nevertheless, such a possibility would require further dedicated testing. Moreover, neither TENT4A nor TENT4B were pulled down with the purified, catalytic human spliceosomes [54].

A recent report using a mammalian cell-free system based on HEK293F cell extracts suggests involvement of TENT4A in miRNP-mediated translational activation of non-adenylated mRNAs [55]. Surprisingly, solely the presence of TENT4A, rather than its poly(A) polymerase activity, seems necessary for this activation. Also, overexpression of TENT4A in this system represses translation of polyadenylated mRNAs. This suggests that TENT4A may also function in a polyadenylation-independent manner. However, the TENT4A clone used by these authors lacked 10 of the 30 N-terminal amino acids reported as highly conserved [52].

### (c) TENT4B, also known as GLD4, PAPD5, TUT3 and TRF4-2

TENT4B is another human orthologue of the yeast Trf4p protein. Initially, TENT4B had been suggested to be involved in uridylation-mediated turnover of replication-dependent histone mRNAs in the cytoplasm [56]. However, this result is controversial and has been challenged by other studies [57,58]. Essentially, human TENT4B has a strong preference for ATP incorporation (in the apparent preference order ATP ≫ GTP > CTP ∼ UTP) with a variety of RNA substrates tested *in vitro*, ranging from oligo(A) and oligo(U) to different tRNAs as well as the 3′ UTR of histone mRNAs [57]. Additionally, examination of TENT4B-EGFP-expressing cells, as well as immunofluorescence analysis, demonstrated its nuclear localization with nucleolar accumulation [49,57]. These findings suggest that TENT4B function in the mammalian nucleus may be similar to that of the TRAMP complex in yeast [59]. Furthermore, and in contrast to yeast Trf4p which requires the Air2p zinc knuckle protein [45,60], human TENT4B does not require a protein cofactor for its polyadenylation activity, thus demonstrating its mechanistical distinction from its yeast counterpart [57].

The analysis of RNAs UV cross-linked to TENT4B *in vivo* revealed that rRNAs are other potential substrates for TENT4B [57]. In mice, TENT4B (but not TENT4A) is involved in the adenylation of aberrant precursor rRNA (pre-rRNA) fragments, leading to their degradation by the nuclear exosome [61]. In line with this finding, TENT4B, ZCCHC7 (hAIR2) and SKIV2L2 (hMTR4) have been identified as components of the human TRAMP complex which, together with the nucleolar exosome possessing EXOSC10 (hRRP6) as a catalytic subunit, are involved in the turnover of pre-rRNA fragments in HeLa cells [49,62–64]. Furthermore, proteomic analysis of TENT4B and ZCCHC7 revealed their interactions with components of the small subunit (SSU) processome, which is the first precursor of the small ribosomal subunit in Eukaryotes involved in the early steps of pre-rRNA processing in the nucleolus [49,50,65], reviewed in [66]. TENT4B, however, is also able to polyadenylate mature rRNAs. In mice, daily oscillations in liver mass arise from regulated changes in the number of ribosomes and their translational activity [67]. Ribosomal protein synthesis is regulated in the phase opposite to the transcription of rRNAs. During daily rest/activity cycles, rRNAs synthesized in excess and not packaged into complete ribosomal subunits are polyadenylated by TENT4B and degraded by the nuclear exosome [67].

TENT4B participates in the maturation of the H/ACA box snoRNAs (small nucleolar RNAs) by adding oligo(A) tails to the last nucleotides remaining after exonucleolytic degradation of the 3′ flanking intron. The oligo(A) tails, together with remaining intron nucleotides, are then removed by poly(A) specific ribonuclease (PARN) resulting in mature and stable snoRNAs [68]. This effect is consistent with a proteomic analysis that detected both C/D box- and H/ACA box-specific proteins in a TENT4B immunoprecipitate [49].

Previous studies have suggested that TENT4B (but not TENT4A) can mediate non-templated 3′ adenylation of some miRNAs in humans [57,69]. In particular, TENT4B-mediated adenylation of the 3′ end of *miR-21-5p* marks it for degradation by PARN. This degradation pathway is disrupted in a wide range of cancers and other proliferative diseases [70]. The oncogenic role of *miR-21-5p* is owing to downregulation of various tumour suppressors. Interestingly, in HER2-amplified tumours *miR-21-5p* trimming is controlled by *miR-4728-3p*-mediated downregulation of TENT4B, leading to high steady-state levels of active *miR-21-5p* [71]. These results suggest that TENT4B itself may be a tumour suppressor.

TENT4B plays an important role in the quality control pathway of human telomerase RNA (hTR) [72]. Mutations in the hTR, the telomerase RNP component dyskerin (DKC1), and PARN can lead to insufficient telomerase activity leading to the *dyskeratosis congenital* (DC) disease. Compromised biding of dyskerin to hTR results in its adenylation by TENT4B, followed by EXOSC10-mediated 3′ to 5′ degradation, as well as decapping by DCP2 and 5′ to 3′ degradation by XRN1. On the other hand, under normal conditions PARN deadenylase competes with TENT4B and by removing oligo(A) tails prevents hTR degradation, maintaining its physiological concentration in equilibrium [72].

A similar model of TENT4B–PARN competition and cooperation has been proposed for Y RNA maturation and degradation [73]. Human Y RNAs are abundant small RNA polymerase III (Pol III)-transcribed RNAs with a role in RNA quality control, histone mRNA processing, DNA replication and damage response [74]. Y RNAs mature in a process involving their adenylation by TENT4B and trimming by PARN and EXOSC10. In the absence of PARN or EXOSC10, the Y RNA possessing oligo(A) tails is degraded by cytoplasmic DIS3L or nuclear TOE1 3′–5′ exoribonucleases [73,75]. Low levels of Y RNAs intensify the effect of PARN depletion on telomere maintenance, leading to the severe phenotype of DC observed in patients carrying PARN mutations [73].

Finally, TENT4B has been reported to act in a pathway affecting the tumour suppressor TP53 (also known as p53) [76]. In this pathway, CPEB binds to the 3′ UTR of *TP53* mRNA and recruits TENT4B, which in a polyadenylation-dependent manner increases *TP53* mRNA stability and thus modulates its translational competence. In turn, the expression of CPEB is regulated by *miR-122*, whose supply depends on its stabilization by TENT2. This may be another piece of evidence of TENT4B acting as a tumour suppressor in human cells. In a recent report from the same laboratory hundreds of other mRNAs whose polyadenylation is regulated by TENT4B have been identified in a genome-wide screen [77]. Several of these mRNAs are involved in carbohydrate metabolism. Depletion of TENT4B reduces *GLUT1* mRNA poly(A) tail length and the level of GLUT1 protein—a major glucose transporter. Similarly, as with *TP53*, TENT4B-mediated stabilization of *GLUT1* mRNA is dependent on CPEB. In addition to this, TENT4B regulates the poly(A) tails of several other mRNAs that are involved in carbohydrate metabolism, including *G6PD*, *PFKFB3*, *PFK-1*, *GK*, *TALDO1* and *ENO1*.

### (d) TENT4A and TENT4B in mixed A/G tailing

**Figure 2.**
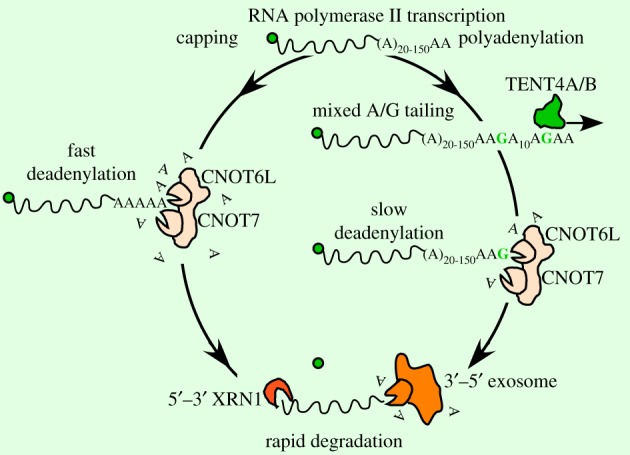
Mixed A/G tailing by TENT4A/B. RNA polymerase II transcribed mRNA is capped and polyadenylated. The poly(A) tail can be additionally tailed with GMP residues by TENT4A/B proteins. The mixed A/G tail (with 1 G incorporated per 10–20 As) is more resistant to the CNOT complex-mediated deadenylation than the pure poly(A) tail as both CNOT6 L and CNOT7 deadenylases fall off their substrates once they encounter a G (a non-A) residue. Ultimately all mRNAs are degraded from both 5′ and 3′ ends following deadenylation and decapping.

Based on similarity, it was expected that TENT4A and TENT4B would at least partially functionally overlap, although their functions have mostly been studied separately so far (figure 1). A recent report proposed an interesting not previously described role of both TENT4A and TENT4B [52]. These enzymes were implicated in polyadenylation of protein-coding mRNAs. However, owing to their slightly promiscuous nucleotide specificity, the enzymes occasionally incorporate GMP within the extended poly(A) tails [52]. Figure 2 depicts the process whereby following TENT4A/B activity, deadenylation of the poly(A) tails is executed by CNOT6 L and CNOT7 within the CNOT deadenylating complex. Deadenylation stops on guanine residues owing to the A-preference of CNOT6 L/7, which ultimately results in an increased frequency of guanine residues on the 3′ ends of mRNA with long poly(A) tails [52]. The process is likely more complex owing to the involvement of other factors including poly(A) binding proteins (PABP) that also participate in the adenylation-deadenylation-driven regulation of poly(A) tails [78–80]. While not explicitly stated in the original study [52], the mixed A/G tailing would likely occur in the nucleus given the nuclear localization of TENT4A and TENT4B [49,50,57]. It is, however, also possible that small fractions of TENT4A/B present in the cytoplasm might also participate in mixed A/G tailing. The mechanism of substrate selection and the general impact of mixed A/G tailing by TENT4A and TENT4B on mRNA metabolism remain to be established. Finally, the importance of mixed A/G tailing in different cellular and tissue contexts and at the organismal levels also requires further experimental confirmation.

### (e) TENT5 proteins

TENT5 comprises a group of four previously unrecognized evolutionarily conserved TENTs also known under the FAM46 acronym (FAMily with sequence similarity 46) in human and mice. These are TENT5A (FAM46A, other synonyms C6orf37, FJL20037), TENT5B (FAM46B, MGC16491), TENT5C (FAM46C, FJL20202) and TENT5D (FAM46D also known as CT1.26, CT112 and MGC26999) (figure 1). TENT5 proteins were initially discovered by an *in silico* study and described as putative poly(A) polymerases [5]. All TENT5 homologues are highly similar in their amino acid sequences and share a common two-domain architecture with (i) an NTase domain distantly related to known NTase domains of other PAPs and TUTases, but comprising well conserved acidic amino acids in its putative catalytic centre and (ii) a poly(A) polymerase/2′–5′-oligoadenylate synthetase 1 substrate binding domain (PAP/OAS1 SBD) [5,81]. The latter is likely involved in substrate RNA binding and stabilization during poly(A) tail formation. Indeed, a recent study confirmed that human TENT5C and TENT5D form RNA–protein complexes and possess poly(A) polymerase activity that depends on the presence of acidic residues within the proteins' NTase domains [82].

In stark contrast to the scarcity of studies describing TENT5 proteins' activity on a molecular level, there are plenty of reports linking mutations in TENT5 proteins to multiple less or more severe conditions.

TENT5A was first described as C6orf37 (Chromosome 6 open reading frame 37), a protein of unknown function with possible relation to human retinal diseases including *retinitis pigmentosa* [83–85]. Furthermore, TENT5A is highly expressed in ameloblast nuclei of tooth germs and may play a significant role in the formation of enamel [86]. Moreover, it has been shown that polymorphism in the second exon of *TENT5A* may be associated with an increased risk of large-joint osteoarthritis [87], which is consistent with severe skeletal abnormalities of *TENT5A* knock-out mice [88]. Finally, loss-of-function mutations in *TENT5A* have been reported in patients suffering from severe, autosomal recessive forms of *osteogenesis imperfecta* [89].

Much less is known about TENT5B. There is only a single report demonstrating upregulation of the protein in refractory *lupus nephritis* [90].

TENT5C is one of the most frequently mutated genes in a B-cell malignancy—multiple myeloma (MM)—following well-known proto-oncogenes of the RAS family [91]. It has been shown, using whole-genome and whole-exome sequencing, that homozygotic or hemizygotic mutations of *TENT5C* are found in 3.4–13% of primary MM cases [91–93]. To date, over 70 different mutations have been identified, most of them frameshift or nonsense mutations [94]. Moreover, deletion of cytoband 1p12, where the *TENT5C* gene is located, was found in about 20% of MM patients and loss of *TENT5C* is associated with limited survival [92]. In a recent study, several MM cell lines bearing *TENT5C* mutations showed significantly reduced growth and lower survival rates of these cells upon expression of wild-type TENT5C [82]. Furthermore, knock-down of TENT5C enhanced proliferation rates of B lymphocytes, thus showing the role of TENT5C in generally suppressing cell growth. Thus, TENT5C likely acts as an onco-suppressor by the specific and robust polyadenylation of a subset of mRNAs mostly encoding endoplasmic reticulum-targeted and secreted proteins [82]. Nevertheless, the specificity mechanism remains unknown. In another study, TENT5C overexpression caused downregulation of transcriptional factors IRF4, CEBPB and MYC, and upregulation of immunoglobulin light chain [95]. Therefore, the effect of mutations of *TENT5C* in MM pathogenesis seems to be caused by the misregulation of the endoplasmic reticulum homeostasis [82,95]. In contrast to some earlier claims of TENT5C being an essential gene whose deficiency would cause embryolethality [96], an independent study demonstrated a lack of major phenotypes in *TENT5C* knock-out mice [82]. Instead, the mice suffered from anaemia likely owing to an abnormality in haemoglobin synthesis that might be connected to TENT5C activity as a growth regulator in the blood cells' B-lineage [82]. Besides MM, the *TENT5C* gene is suggested to play a role in the pathogenesis of other tumours [97–99]. *TENT5C* expression is significantly lower in hepatocellular carcinoma than in normal liver tissue [99]. Moreover, *TENT5C* is upregulated in response to norcantharidin (an antimetastatic drug used in hepatocellular carcinoma). Upregulation of *TENT5C* causes reduction of cancer cell migration and invasion [98]. A similar effect is observed in gastric cancer—in cancerous tissues, a significant reduction of *TENT5C* levels is observed. Low *TENT5C* levels are associated with higher risk of recurrence after gastric resection and generally poor prognosis [97]. Finally, mutations in *TENT5C* also appear to be related to autism [100].

TENT5D dysfunction might also be related to autism as TENT5D is overexpressed in the cerebral cortex of mice with autism-like behaviours [100]. Finally, in humans, antibodies against TENT5D are present exclusively in the serum of patients suffering from testis and lung tumours [101].

### (f) MTPAP, also known as FLJ10486, mtPAP, PAPD1, SPAX4 and TENT6

MTPAP is unique among all the other TENTs as it is localized exclusively in mitochondria and is the only known ncPAP known to polyadenylate mitochondrial RNAs [102,103]. *In vitro*, MTPAP can use all four nucleotides as substrates, although the strongest activity is observed with both ATP and UTP [104]. Interestingly, structural and biochemical analysis suggests that the enzyme is active only as a dimer [104]. MTPAP does not seem to rely on RNA-binding proteins for substrate recognition, but some proteins could enhance its activity. For instance, LRPPRC/SLIRP, a mitochondrial RNA-binding complex, enhances the polyadenylation of mitochondrial mRNAs (mt-mRNAs) by MTPAP *in vitro* [105,106]. Furthermore, MTPAP might interact with a complex formed by mitochondrial RNA helicase SUV3 and exoribonuclease PNPase to regulate the length of mt-mRNA 3′ poly(A) depending on the inorganic Pi/ATP ratios and so in response to cellular energy changes [107]. In contrast to cellular cytoplasmic mRNAs, the poly(A) tails in mt-mRNAs serve different functions. In mammalian mitochondria, a majority of the mtDNA-encoded mRNAs (in humans 7 out of 13) have incomplete stop codons represented only by the U or UA. MTPAP adds AMP residues to the 3′ end of mt-mRNAs forming an oligo- or poly(A) tail and simultaneously generating the proper UAA stop codon (reviewed in [108–110]). MTPAP also plays an important role in the maturation of mt-tRNAs. In human mitochondria the genes coding for tRNA Tyr and tRNA Cys overlap by one nucleotide, which results in an incomplete tRNA Tyr precursor lacking the 3′-terminal adenosine. This precursor is a substrate for MTPAP, which may add one or more AMPs to its 3′ end. If only one AMP is added, the tRNA Tyr precursor becomes a substrate for CCA addition and further acetylation. In case of MTPAP adding an oligo(A) tail, either the 3′–5′ deadenylase PDE12 or the endonuclease RNase Z removes the excessive nucleotides, producing a substrate for CCA addition [111,112].

The role of mitochondrial polyadenylation in RNA stability, turnover and translation remains an open question and is discussed elsewhere [108–110]. In two different studies, it has been observed that the silencing of MTPAP leads to the shortening of mt-mRNAs' poly(A) tails [102,103]. However, in one of these reports no changes in a steady state level of mt-mRNAs or their protein products have been observed [103], whereas according to another study, knock-down of MTPAP decreases the stability of the *CO1*, *CO2*, *CO3* and *ATP6* mRNAs, but has no effect on the *ND3* mRNA [102]. Interestingly, a homozygous N478D mutation in MTPAP also results in shorter poly(A) tails for all mt-mRNA transcripts tested, but its effect on stability is transcript-dependent [106].

Two other functions have been proposed for MTPAP. First, it has been suggested to oligouridylate histone mRNAs, inducing their degradation [56]. Another study proposed that MTPAP may be involved in adenylation of some miRNAs [113]. However, since MTPAP is an exclusively mitochondrial protein, these functions are controversial.

There are reports linking mutations in *MTPAP* to some genetic disorders. Mutation N478D in the so-called fingers domain within a very conserved region of the protein is associated with an autosomal-recessive disease—spastic ataxia with optic atrophy [114]. In this condition the poly(A) tails of mt-mRNAs are significantly shorter in all homozygous individuals as compared to hemizygous carriers and healthy individuals [106,114]. Further work established that the homozygous N478D mutation also causes cellular radiosensitivity and persistent DNA double-strand breaks [115]. Another mutation in *MTPAP*, D39G, was found to be associated with extreme obesity in cattle [116]. Molecular mechanisms leading to the observed disorders remain elusive.

## 3. TUTases and uridylation

Uridylation is a common phenomenon reported for the majority of RNA species in a mammalian cell. In this process one to 20+ uridines are appended to the RNA 3′ end by either of three confirmed TUTases: TENT1 (U6 TUTase), TUT4 or TUT7. While TENT1 is a nuclear enzyme, TUT4 and TUT7 localize in the cytoplasm, which in turn defines their substrate RNAs. In general, in mammals, uridylation has been linked with the biogenesis of certain RNAs and with reduced stability of the uridylated RNAs. Here we describe the major findings with each of the TUTases, the differential impact of uridylation on different RNA classes and the global role of uridylation in mammals, concluding with the newest findings. See also reviews in this issue by Zigackova and Vanacova [117] and De Almeida *et al*. [118] on uridylation in other organisms.

### (a) TENT1, also known as RBM21, TUT1 and U6 TUTase

TENT1 is a protein highly evolutionarily conserved among vertebrates. It is widely expressed in all human tissues and a decrease in its level in cell lines leads to reduced proliferation rates and viability [119,120]. In line with this, most high-throughput genome-scale RNAi or CRISPR-based screens identified *TENT1* as an essential fitness gene [121–124]. TENT1 is dominantly localized in the nucleoplasm and nuclear speckle body-like structures and/or nucleolus [125,126]. Its localization in the nucleolus depends on an interaction with the NMP1 nucleolar protein and is reduced by ubiquitination by the Cullin-RING ubiquitin ligase complex subunit—KLHL7 protein [125]. TENT1 comprises several domains, whose organization is different from TUT4 and TUT7 (figure 1) [127,128]. Starting from the N-terminus, TENT1 contains: a putative zinc finger (ZF) domain, an RNA recognition motif (RRM), a so-called ‘palm’ with a proline-rich region (PRR) insertion, so-called ‘fingers’ and a kinase associated-1 (KA-1) RNA-binding domain and a nuclear localization signal (NLS) [128]. The ZF, RRM and KA-1 domains bind RNA, thus the protein likely does not require additional protein cofactors for RNA binding [128,129]. The mechanistic model deduced from the TENT1 crystal structure implies that the enzyme, by open-to-close state transitions, adds several UMPs to the RNA's 3′ end, which becomes compressed within the enzyme's active pocket. Once the RNA can no longer translocate to the active site it dissociates [128]. The PRR region splits the PAP domain and can be phosphorylated by casein kinase I (CKI) isoforms α and ɛ, which modulates the enzyme's activity [130]. Besides RNA binding, the KA-1 domain can also bind phospholipids, and so it might play a role in the postulated PtdIns-4,5(phosphatidylinositol-4,5-bisphosphate)-P2-dependent activation of TENT1, as described below [128].

#### (i) TENT1 in U6 small nuclear RNA (snRNA) biogenesis

TENT1 was first discovered as an enzyme crucial in biogenesis of U6 snRNA responsible for the catalysis of pre-mRNA splicing [117,131–133]. The nascent U6 snRNA transcript contains a tract of four uridines (Us) at the 3′ end that serves as a termination signal for Pol III [134]. To become functional, U6 snRNA requires further post-transcriptional 3′ end maturation involving two opposite activities: oligouridylation carried out by TENT1 [117,131–133] and subsequent exonucleolytic trimming executed by the USB1 protein, whose activity additionally leads to formation of a 2′–3′ cyclic phosphate at the 3′ end—a hallmark of U6 snRNA [135–139]. As a result, the majority of mature human U6 snRNAs contain five terminal uridines and a 2′–3′ cyclic phosphate moiety that protects them from oligouridylation-mediated destabilization [118,134,140]. The presence of the terminal U-rich stretch is also crucial for U6 snRNA function in pre-mRNA splicing. Briefly, the uridine-rich 3′ tail constitutes a binding platform for the heteroheptameric Lsm2-8 protein complex that, cooperatively with the Prp24p protein, facilitates the annealing of U6 and U4 snRNAs during U4/U6 di-snRNP formation, as shown for the yeast U6 snRNP [141–144]. Thus, TENT1 contributes to increased stability of U6 snRNA molecules and indirectly to pre-mRNA splicing regulation.

#### (ii) TENT1 as an adenyltransferase. The Star-PAP

There is some controversy regarding the role of TENT1 in nuclear adenylation of mRNAs in response to stress conditions. A decade ago TENT1 was reported as a highly processive nuclear speckle-targeted and PtdIns4,5P2-regulated nuclear poly(A) polymerase (Star-PAP) *in vitro* [126,145,146]. It was suggested that TENT1 serves as a biosensor for the transduction of stress signals within the cell nucleus through targeting mRNAs involved in oxidative stress response (*HO-1* and *NQO-1*) and mRNA of a pro-apoptotic gene *Bcl-2 interacting killer* (*BIK*) [126,145,146]. It was postulated that TENT1 interacts with CPSF-73, CPSF-160 of the Cleavage and Polyadenylation Specificity Factor and several other proteins, leading to the formation of poly(A) tails, instead of the canonical poly(A) formation pathway employing PAPα/γ, and thus specifically regulating the supply of selected mRNAs [126,129,147,148]. However, recent structural studies of nucleotide recognition by TENT1 revealed a superior coordination of the uracil base by hydrogen bonding with two conserved Asn and His residues and some stacking interactions within the enzyme's nucleotide binding pocket, while ATP is stabilized by just a single hydrogen bond [128]. The arrangement of core TENT1 domains is topologically homologous to the yeast Cid1 uridyltransferase (but also to the human MTPAP). In line with this, biochemical *in vitro* activity assays showed that TENT1-mediated uridylation of U6 snRNA-u4 and *3′-UTR-HO1* transcripts is tens of times more efficient than adenylation [128], which is in disagreement with a previous report showing higher ATP specificity of TENT1 in *in vitro* extension of *A15* and *A44* oligonucleotides [126]. Nevertheless, the substrates used in the two experimental set-ups were significantly different, i.e. poly(A) RNAs in [126] and *HO-1* 3′ UTR without 3′ adenines in [128], which might have influenced the observed preferences. Given the highly complex nature of the 3′ end regulatory networks (figure 2) it also cannot be ruled out that TENT1 activity changes *in vivo* owing to its allosteric or structural transitions under specific physiological conditions, such as oxidative stress, or through specific interacting proteins. Interestingly, TENT1 interacts also with nuclear PIPKIα and PKCδ kinases, which by phosphorylation might change its substrate preference from UTP to ATP [126,146,147]. Finally, some recent reports also indicate that TENT1 interacts with both the Perlman syndrome 3′–5′ exoribonuclease DIS3L2 and Argonaute2 in an RNA-dependent manner, contributing to the regulation of miRNA abundance by uridylating RISC-bound miRNAs and inducing their degradation [113,149–151]. This process would likely happen in the cytoplasm and thus it constitutes another controversy regarding TENT1.

### (b) TUTases (TUT4 and TUT7) in cytoplasmic RNA uridylation

TUT4 (also known as KIAA0191, PAPD3, TENT3A and ZCCHC11) and TUT7 (also known as FLJ13409, KIAA1711, PAPD6, TEBT3B and ZCCHC6) share significant sequence similarities and TUT7 is thought to have evolved as a result of TUT4 duplication [152]. Both TUT4 and TUT7 are large proteins (in human approximately 185 and 171 kDa, respectively) and comprise catalytic ribonucleotidyltransferase domains with a conserved DDD triad in their catalytic centres and, interestingly, non-catalytic NTr-like domains lacking critical catalytic aspartate residues (figure 1). Important are four zinc finger/knuckle domains of C2H2 (one, ZF) and CCHC (three, ZK) types scattered within the proteins' bodies. The ZF and ZKs might act as protein–RNA and protein–protein interaction platforms. Indeed, the first of these motifs from the protein's N-terminus (ZF) has been demonstrated to interact with the LIN28a protein [127,153] and the third has been shown to be involved in the stabilization of the growing oligouridine tail during uridylation of the pre-let-7 miRNA precursors [127]. TUT4 comprises two additional domains—one on its N-terminus and one on its C-terminus. These domains seem irrelevant for uridyltrasferase activity but might play some other yet undiscovered roles [127,153]. Most reports suggest redundant functions of TUT4 and TUT7, however, depending on cellular model or tissue context, these enzymes might also perform different functions.

#### (i) Oligo- and monouridylation in pre-miRNA regulation—a double faceted effect of TUTases

Initially, TUTases were characterized for their role in uridylating precursors of the *let-7* miRNA family. These miRNAs are involved in cell differentiation and deregulated in cancer development (reviewed in [154,155]). In non-differentiated cells the LIN28a protein is expressed. It specifically binds miRNA precursors and a TUTase, promoting processive oligouridylation of the precursor miRNA, which ultimately precludes its processing by DICER and leads to degradation of the oligouridylated *pre-let-7* [156–163]. In contrast to that, in differentiated cells LIN28a is not expressed [42,164]. In its absence, TUT4/7 add prevalently just a single uridine to the 3′ end of the *pre-let-7* RNAs. In fact, miRNA precursors fall into two families regarding their 3′ end: it is either a 2-nucleotide 3′ overhang (group I of prototypical pre-miRNA) or just a single nucleotide 3′ overhang (group II). Since DICER requires a 2-nucleotide 3′ overhang for processing of the pre-miRNA into mature miRNA, group II pre-miRNA comprising a majority of *let-7* family RNAs cannot be processed [42]. However, once monouridylated, the group II pre-miRNAs are conveyed to the later steps of their maturation [42,43]. Thus, a single protein LIN28a provides a crucial discriminatory mechanism for either oligo- or monouridylation and their respective effects in blocking or promoting microRNA maturation. Small molecules inhibiting LIN28a-*pre-let-7* interactions have recently been published providing a foundation for the treatment of LIN28a-induced disorders, mainly different cancers ([165] and references therein).

Apart from LIN28a, another protein—TRIM25—has been implicated in pre-miRNA oligouridylation [164]. TRIM25 might act as a *pre-let-7*-specific activator of LIN28a/TUT4-mediated uridylation. In mammals also mature miRNAs are globally and/or specifically uridylated under a variety of conditions including normal growth, differentiation, in response to either dynamic environmental changes, accompanying viral infection or in maintenance of steady-state naive T cells [113,149,166–170]. As a result of uridylation miRNAs might lose their regulatory potential and are destined for degradation.

Experimental evidence gathered so far and recent structural information on the pre-miRNA-LIN28a-TUT4 ternary complex revealed a processivity mechanism for uridylation wherein all components of the ternary complex contribute to stabilization of the TUTase-RNA interactions [127,171]. Furthermore, once a few uridines are appended, the ZK 2 domain in the TUTase engages the growing oligo(U) tail in U-specific interactions that altogether assure enough stability for further processive oligo(U) addition by the TUTase [127]. The structure also suggests the way in which the TUTase discriminates between group I and group II pre-miRNA, which relies on specific binding of group II miRNA precursors to the enzyme in a pre-catalytic state. Once monoU has been added, the newly formed 2-nucleotide overhang positions the RNA in a post-catalytic state reinforcing RNA dissociation. A similar non-favourable positioning in the post-catalytic state accounts for a lack of extension on the group I miRNA precursors in the absence of LIN28a [127]. In the absence of LIN28a TUT4/7 uridylate exposed 3′ ends in a distributive manner without the need for a protein cofactor [43,172].

#### (ii) Uridylation in replication-dependent histone mRNA clearance

It is currently acknowledged that TUT7 and to a lesser extend TUT4 are responsible for uridylating histone mRNAs [57,58,173]. Histone mRNAs form a distinctive group of mammalian mRNAs, since they possess a stabilizing 3′ stem-loop instead of a poly(A) tail [174,175]. The availability of histone mRNAs in a cell is tightly regulated so that their expression keeps pace with DNA replication in the S phase. The tight regulation is important since if expressed in other cell growth phases histones interfere with several cellular processes leading to severe cytotoxicity [176]. The tight regulation takes place both on transcriptional and post-transcriptional levels to ensure histone supply at the onset of S phase and their rapid clearance once replication is completed. In mammals, transcription of histone mRNAs changes 5–6 fold during cell cycle [177,178], thus the pivotal role in regulation of histone mRNA supply is their uridylation-dependent clearance [56]. For details on histone mRNA regulation see a comprehensive review by Marzluff & Koreski [179] and a review by Stacie *et al*. [180].

#### (iii) mRNAs—general importance of uridylation in apoptosis, oocyte and embryo development

With the development of TAIL-Seq, a tool for global 3′ terminome analysis [181], it became apparent that not only replication-dependent histone mRNAs but also mRNAs acquiring poly(A) tails are uridylated, though to different extents and depending on their poly(A) tail lengths. While for some mRNA species nearly 50% possessed 3′ non-templated uridines, for some others only a minor fraction (less than 2%) were uridylated [182]. This discrepancy might have resulted from either specific uridylation or less effective degradation of some mRNAs, but the mechanistic explanation of either possibility requires further testing. In general, uridylation occurs frequently with mRNAs possessing short poly(A) tails of less than 20 As [182]. Uridylation levels correlate with decreased mRNA stabilities [182] that at least partially result from removal of uridylated mRNAs by the DIS3L2 3′–5′ exoribonuclease [140,180]. Uridylated RNAs are also likely removed by the 5′–3′ decay, since uridylation induced decapping, as shown in studies with uridylated reporter RNAs in human cellular extracts [184]. It is likely that similarly to the situation in fission yeast, uridylated RNAs are bound by LSM1-7, decapped by the decapping complex (involving several protein components; see [185] for a review) and degraded by the XRN1 5′–3′ exoribonuclease [186]. Furthermore, abortive initiation of mRNA transcription by Pol II leads to the production of so-called transcriptional start-site-associated RNAs—TSSs. These are pervasively oligouridylated and undergo uridylation-dependent clearance by DIS3L2 [187,188].

There is no doubt that uridylation is common in mammalian cells. However, how essential is the modification? Is uridylation just a fine-tuning mechanism in RNA decay that if missing can be replaced by other pathways, or does uridylation play an indispensable role and, if so, is it spatially or temporarily restricted? A study by Thomas *et al*. [189] highlighted the global importance of uridylation in apoptosis. Apoptosis is a complex process involving multiple regulatory mechanisms that occur in a step-wise manner. On the level of RNA, initial apoptotic mitochondrial outer membrane permeabilisation (MOMP) induces a global degradation of translation-competent mRNAs but not of non-coding RNAs at the onset of apoptosis [189,190]. The global mRNA decay is induced by TUT4/7-mediated uridylation and executed by DIS3L2 3′–5′ exoribonuclease [189]. Simultaneously, pre-mRNA splicing and RNA nuclear export are inhibited, which prevents the production of stress-responsive factors [191] and precedes phosphatidylserine externalization and DNA fragmentation. In fact, knock-down of TUTases or DIS3L2 leads to anti-apoptotic effects and increases survival of cells exposed to apoptotic stimuli [180,189].

Recent evidence identifies another crucial role of TUTases and uridylation in gametogenesis and early development. These results come from a study with TUT4/7 conditional knock-out (cKO) mice that demonstrated that TUTases are dispensable in adult animals since their lack does not lead to global transcriptome changes in somatic cells [35]. Instead, the TUT4/7-mediated uridylation regulates the maternal protein-coding transcriptome in developing oocytes and is indispensable to complete meiosis I and for generation of functional oocytes as well as for early embryo development following fertilization, as the fertilized *TUT4/7cKO* did not develop past two-pronuclei stage [35]. The early embryo is transcriptionally inactive and relies on maternally deposited transcripts. Thus, the regulation of transcript supply implies mostly poly(A) tail length adjustments and uridylation-induced degradation of certain transcripts at the completion of a programmed developmental stage. The global effect of TUTases and uridylation in oogenesis (and likely embryogenesis) apparently requires oligouridine tails as the ratio of oligo- to monouridylated RNAs is significantly higher in oocytes than in other investigated mouse cell types and tissues [35]. TUT4/7 depletion resulted in a deregulation of a group of transcripts that were upregulated in *TUT4/7cKO* oocytes and lacked oligouridine tails present in the control *TUT4/7CTL* oocytes, while a pool of transcripts remained unchanged between these test conditions. It seems awkward, however, that only a minor fraction of less than 2% of transcripts was found uridylated in the control oocytes (and other cells) in this study. The above observations thus suggest that the specificity of TUT4/7-mediated uridylation is important, leading to elimination of only a strictly defined cohort of transcripts and thus likely allowing for smooth maternal-to-zygotic transition. Such conclusions were only recently made also for *X. laevis* and zebrafish [192]. By using a morpholino-induced conditional depletion of TUT4/7 homologues in *X. laevis* and zebrafish embryos it has been demonstrated that the TUT4/7-mediated uridylation is at the onset of maternal transcriptome clearance during maternal-to-zygotic transition at 4–6 h post fertilization [192]. On the basis of these reports it becomes apparent that TUTases are especially needed in oogenesis and at early stages of embryo development.

#### (iv) Uridylation of snRNA, tRNA and other RNA polymerase III transcripts

TUTases play roles in the regulation of a cohort of other RNA species in the cytoplasm. Among them are snRNAs that constitute integral parts of the pre-mRNA splicing catalysing spliceosome [193]. Four of these RNAs—*U1*, *U2*, *U4*, *U5*—are transcribed by RNA polymerase II and one—*U6* snRNA—by RNA polymerase III, and all undergo a series of specialized processing steps both in the nucleus and in the cytoplasm [137,194]. Misprocessed snRNAs are uridylated by the TUTases and destined for DIS3L2-mediated decay [140,188,195]. Initially, the TUT-DIS3L2 pathway was considered in nuclear snRNA processing and biogenesis. However, this possibility was ruled out [140], which was later corroborated by the discovery of a nuclear snRNA processing 3′–5′ exoribonuclease—TOE1 [196].

In a CLIP assay with a mutant DIS3L2 (D391N), uridylation sites have been found within bodies of all mature rRNA species: *28S*, *18S*, *5.8S* and the Pol III-transcribed *5S* rRNAs, and less so, but also present, in the so-called ETS and ITS parts of the rRNA precursor [188]. The identified fragments most likely represent degradation intermediates, thus strongly suggesting that rRNAs are also targets of (most likely) TUT4/7-mediated uridylation and rely on subsequent degradation by DIS3L2 [188].

Transcription by Pol III complements mammalian RNAs with diverse short and usually highly structured non-coding RNAs [197,198]. Importantly, all these transcripts end in 4–5 Us, which is a termination signal for Pol III-mediated transcription. Recent evidence confirms the generality of the cytoplasmic uridylation-induced DIS3L2-executed RNA decay in regard to many Pol III transcripts including U6 snRNA, snoRNA, tRNA, *Y* and *vault* RNA, *Rmrp*, *7SL*, *BC200* and several others [140,188,195]. Transfer RNAs (tRNAs) are likely some of the most notable regulated RNAs as they play an essential function in protein biosynthesis. In their CLIP study, Ustanienko *et al.* [188] showed mapping to tRNA truncated within the T-loop or to the 3′-end tRNA trailers, which implied that uridylation-induced DIS3L2 decay involves misprocessed extended forms of tRNAs and likely might also regulate properly processed tRNAs. *Y* and *vault* RNA (*VTRNA*) are short RNAs that form RNP assemblies in the cytoplasm. In fact, *VTRNA*s form likely the biggest known human cytoplasmic RNPs with a mass of approximately 13 MDa and overall dimensions of 40 × 40 × 70 nm [199]. While Y RNA likely play a role in DNA replication and RNA processing [74], *VTRNAs* have been linked to multidrug resistance and anti-apoptotic effects in cancer cells [200]. Moreover, both ncRNA types might serve as precursors for the generation of short RNA, svRNA and Ys RNA, which likely act in post-transcriptional regulation of some mRNAs [201,202]. The importance of these RNAs in cells has not been firmly established. Nevertheless, they are among the most prominent TUT4/7-DIS3L2 substrates [140,188,195]. Another Pol III transcript regulated by uridylation and DIS3L2-mediated decay is *Rmrp*. Its function in mammalian cells is not clear but mutations in human *RMRP* gene lead to *cartilage–hair hypoplasia* (CHH), manifesting in a few serious deficiencies [203]. Imprecise 3′ ends of *Rmrp* RNAs have been found heavily oligo- and polyuridylated in DIS3L2 co-immunoprecipitates, with as many as 26 3′ uridines and a median length of 12 uridines [195]. Interestingly, since Pol III-transcribed ncRNAs were the dominant fraction of RNAs enriched in DIS3L2 co-IPs, the authors proposed that ncRNAs are prime targets of the uridylation-induced DIS3L2-executed decay [195]. This, however, might at least partially result from particular features of the short ncRNAs, namely: (i) naturally occurring 4–5 Us at their 3′ ends, which might remain free from base-pairing interactions and protrude from RNPs [140,187,188,195]; and (ii) their stable structures that might effectively stall DIS3L2 on these substrates (Warkocki *et al*. 2016, unpublished). *In vitro* reconstitution of TUTase activity and DIS3L2-mediated RNA degradation assured that at least in the case of the tested tRNA, *Y* and *vault* RNAs and *Rmrp* the concerted uridylation-induced 3′–5′ decay does not require other protein factors besides a TUTase and DIS3L2 [140,188,195].

#### (v) Uridylation of RNA viruses and human LINE-1 retrotransposons

**Figure 3.**
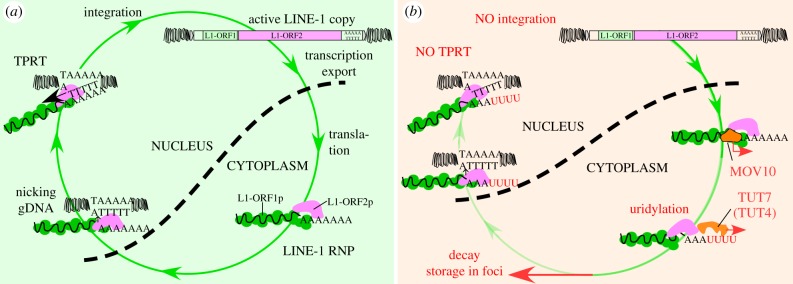
Involvement of TUT4 and TUT7 in LINE-1 retrotransposon restriction. Panel (*a*) highlighted in green shows the LINE-1 retrotransposon life cycle. An active LINE-1 copy is transcribed by RNA polymerase II into a *bicistronic* LINE-1 mRNA and exported into cytoplasm. There LINE-1 proteins—L1-ORF1p (a LINE-1 mRNA chaperone) and L1-ORF2p (a dsDNA nickase and reverse transcriptase) are translated, leading to the formation of a LINE-1 RNP. The RNP is re-imported into the nucleus where the L1-ORF2p nicks genomic DNA, releasing a short (approx. 4–6 nt) stretch of dT. This base-pairs with the LINE-1 mRNA poly(A) tail and so becomes a primer for reverse transcription (known as target-primed reverse transcription—TPRT). Following TPRT, a new LINE-1 copy is ultimately inserted into the genomic DNA by a not well understood mechanism. Panel (*b*) highlighted in pink shows a postulated model of a cooperative activity of MOV10 helicase/RNPase protein and TUT4/TUT7 leading to restriction of LINE-1 retrotransposition. Following the export of LINE-1 mRNA into cytoplasm, MOV10 actively removes L1-ORF1p from LINE-1 mRNA 3′ end and exposes it to enzymatic activity including uridylation by TUT7 and/or TUT7. Uridylated LINE-1 mRNAs undergo decay in the cytoplasm but some LINE-1 mRNPs re-enter the nucleus. There, however, the 3′ uridines do not base-pair with the exposed genomic oligo(dT), thus the reverse transcription cannot commence. As a result, LINE-1 retrotransposition is efficiently restricted by a multi-layered mechanism.

It has been demonstrated that exogenous RNAs of viral origin are also heavily uridylated with as many as a few tens of uridines appended to their 3′ ends [204]. Indeed, a recent report demonstrated that RNA viruses constitute an important target for uridylation by TUT4/7 in *C. elegans*, human A549 cells and murine fibroblasts [205]. While in wild-type cells a significant fraction of viral RNAs was uridylated, in cells lacking TUTases this fraction was reduced to nearly 0 and the percentage of infected cells was also significantly higher than in the wild-type cells. Thus the authors concluded that uridylation by TUT4/7 constitutes a defence mechanism against infections by RNA viruses [205]. Last but not least, a recent study demonstrated a potent multi-layer restriction of human LINE-1 retrotransposons by uridylation [206]. LINE-1 is a group of vertebrate retrotransposons that in humans constitute nearly 17% of the entire genome [207,208]. They proliferate by a copy-and-paste mechanism involving transcription and reintegration into a new site within the genomic DNA by a so-called target-primed reverse transcription (TPRT) mechanism potentially leading to *de novo* mutations in the germline owing to the temporal loss of epigenetic marks that silence LINE-1 in somatic cells [189]. Nevertheless, LINE-1s are also expressed in some somatic cells, especially neurons [209–211]. They are also expressed in cancers [212]. In contrast to other protein-coding mRNAs, uridylation of LINE-1 mRNA not only enhances degradation of otherwise extremely stable LINE-1 mRNAs but also, and most importantly, it might block initiation of reverse transcription during TPRT, which under normal conditions requires base-pairing of the poly(A) tail of LINE-1 mRNA with a short oligo(dT) stretch released from genomic DNA (figure 3) [172,206,213]. Such base pairing cannot be achieved between the oligo(U) tail of mRNA and genomic oligo(dT) [206]. Importantly, TUT4 and TUT7 show slightly different effects on LINE-1 mRNA steady-state levels and stabilities that likely results from TUT4, but not TUT7, enrichment in cytoplasmic *foci* [206].

Biochemical investigations demonstrated that structured RNAs with their 3′ end involved in base-pairing acquire none or shorter U-tails than non-structured substrates or substrates with their 3′ ends clearly protruding from the main RNA body [140,214]. Thus it seems that a mechanistic prerequisite for uridylation might involve resolving secondary and tertiary structures or removal of proteins (like PABPC [182]), both of which occlude 3′ ends and prevent uridylation of some physiological TUT substrates. One could expect helicases and RNPases (proteins destabilizing RNA–protein interactions) to functionally cooperate with the TUTases by removing proteins or resolving secondary and tertiary structures to promote uridylation. Indeed, such a functional cooperativity mechanism involving MOV10 helicase and TUT4/7 has been recently proposed for uridylation of LINE-1 mRNAs that otherwise are tightly packed and protected from external enzymatic activity by a shell formed by multiple copies of the LINE-1 L1-ORF1p chaperone protein (figure 3) [206].

## 4. Conclusion

Eleven mammalian TENTs play important roles in post-transcriptional gene expression regulatory mechanisms acting in nucleus, cytoplasm and mitochondria. They mainly control the stability of RNA species. Cytoplasmic ncPAPs (TENT2, TENT5) stabilize substrate mRNAs, while polyadenylation by the nuclear counterparts (TENT4A/B) seems to have mixed effects. Such enzymes can induce exosome-mediated decay or, because of their promiscuous nucleotide specificity with substantial incorporation of GMP residues within the poly(A) tails of mRNA molecules, they can stabilize mRNAs when they are exported into the cytoplasm. Cytoplasmic TUTases (TUT4/TUT7) mostly induce RNA decay, while nuclear TUT1 stabilizes U6 snRNA. Importantly, although in some cases knowledge about their role and mechanism of action is already substantial, in many cases, TENT5 enzymes for instance, we are just at the beginning of the journey. It is also important to point out that there are several substantial controversies in the field, some of which were described herein. Thus, further research is clearly needed to understand how TENTs regulate gene expression in mammals with a closer look at the subcellular, cellular, tissue and developmental stage contexts.

## Data accessibility

This article has no additional data.
